# Prevention of Atrial Fibrillation: Putting Proteostasis Derailment Back on Track

**DOI:** 10.3390/jcm12134352

**Published:** 2023-06-28

**Authors:** Preetam Kishore, Amelie C. T. Collinet, Bianca J. J. M. Brundel

**Affiliations:** Physiology, Amsterdam UMC, Vrije Universiteit, Amsterdam Cardiovascular Sciences, Heart Failure and Arrhythmias, 1081 HZ Amsterdam, The Netherlands; p.kishore@amsterdamumc.nl (P.K.); a.c.t.collinet@amsterdamumc.nl (A.C.T.C.)

**Keywords:** atrial fibrillation, proteostasis, DNA damage, heat shock proteins, mitochondria, histone deacetylases, autophagy, endoplasmic reticulum stress, microtubules, recovery, electropathology

## Abstract

Despite the many attempts to treat atrial fibrillation (AF), the most common cardiac tachyarrhythmia in the Western world, the treatment efficacy of AF is still suboptimal. A plausible reason for the suboptimal efficacy is that the current treatments are not directed at the underlying molecular mechanisms that drive AF. Recent discoveries revealed that the derailment of specific molecular proteostasis pathways drive electrical conduction disorders, contractile dysfunction and AF. The degree of this so-called ‘electropathology’ corresponds to the response to anti-AF treatment. Hence, to develop effective therapies to prevent AF, understanding the molecular mechanisms is of key importance. In this review, we highlight the key modulators of proteostasis derailment and describe the mechanisms that explain how they affect electrical and contractile function in atrial cardiomyocytes and AF. The key modulators of proteostasis derailment include (1) exhaustion of cardioprotective heat shock proteins (HSPs), (2) excessive endoplasmic reticulum (ER) stress and downstream autophagic protein degradation, (3) histone deacetylase 6 (HDAC6)-induced microtubule disruption, (4) activation of DNA damage-PARP1 activation and NAD^+^ axis and (5) mitochondrial dysfunction. Furthermore, we discuss druggable targets within these pathways that are involved in the prevention of proteostasis derailment, as well as the targets that aid in the recovery from AF. Finally, we will elaborate on the most favorable druggable targets for (future) testing in patients with AF, as well as drugs with potential benefits for AF recovery.

## 1. Introduction

Until today, selecting an efficient management strategy for atrial fibrillation (AF), the most common and age-related cardiac arrhythmia in Western and specific Eastern parts of the world, has been challenging [1]. The main reason for suboptimal AF management is that the exact root causes driving AF are not completely understood; therefore, effective AF diagnostic instruments and therapies are lacking. It has been acknowledged that AF may severely affect the patient’s quality of life as it is associated with serious comorbidities, resulting in increased morbidity and mortality, including cognitive impairment, stroke and heart failure [2]. As the life expectancy of the worldwide population is increasing, a steep rise in AF incidence among the general population is observed, making AF a pressing public health issue.

At present, the treatment modalities of AF follow the ‘one-size-fits all approach’ and existing therapies are only moderately effective. In addition, they do not prevent AF progression from recurrent intermittent episodes (paroxysmal) to (longstanding) persistent and finally permanent AF. Although invasive isolation therapy of pulmonary veins is promising in the early stages of AF, up to 70% of the patients with persistent AF reveal AF recurrences within 1 year and require multiple procedures [3,4]. The currently available pharmacotherapy of AF, originating from the 1960s, targets ion channels and fails to prevent the progression of AF in 85% of the cases. Moreover, its usage is limited by side effects that can even potentially be severe and life-threatening [5]. Furthermore, the individual responsiveness of a patient to AF therapy cannot be sufficiently predicted, forcing treatment selection to be based on ‘trial-and-error’. Despite the fact that several studies identified novel druggable targets that are directed at the root causes of AF [6,7,8,9], new drugs are not available. This absence of effective AF therapies runs in parallel with the absence of knowledge on the mechanisms of AF pathogenesis in individual patients. Therefore, at present, there is an increasing interest in the dissection of the root causes of AF to develop novel mechanism-based personalized treatment modalities.

Reports from both experimental and clinical AF studies showcase the so-called ‘electropathology’ as a key driver of AF. Electropathology is defined as electrical conduction disorders, and consequently contractile dysfunction caused by molecular changes in the atrial cardiomyocyte and tissue that drives AF initiation and perpetuation. Emerging pathways for molecular changes include derailment in protein homeostasis (proteostasis). Key modulators of proteostasis derailment include (1) the exhaustion of cardioprotective heat shock proteins (HSPs), (2) excessive endoplasmic reticulum (ER) stress and downstream autophagic protein degradation, (3) histone deacetylase 6 (HDAC6)-induced microtubule disruption, (4) activation of DNA damage-PARP1 activation and NAD^+^ axis and (5) dysfunction in mitochondrial activity. Modulators of these crucial pathways could indicate potential druggable targets that could aid in the attenuation of AF progression, prevent AF recurrence and promote recovery.

In this review paper, we focus on the key molecular pathways driving proteostasis derailment in atrial tissue and cardiomyocytes, identify druggable targets and discuss the most favorable targets for (future) testing in clinical AF trials.

## 2. Proteostasis Derailment Pathways Driving AF

### 2.1. Exhaustion of Heat Shock Protein Levels

**Figure 1:**
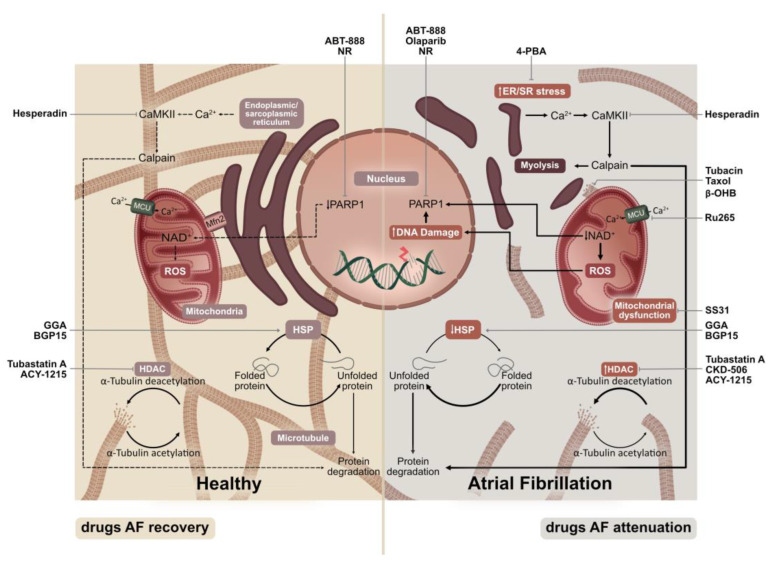
Overview proteostasis derailment and druggable targets to attenuate and recover from AF. Atrial fibrillation (AF) causes derailed proteostasis due to loss in protein quality control by exhaustion of (1) the heat shock proteins (HSPs). Induction of HSPs by geranylgeranylacetone (GGA) or BGP-15 attenuates and even reverts proteostasis derailment and AF promotion. (2) Autophagic protein degradation is induced by increased endoplasmic/sarcomplasmic reticulum stress (ER/SR; ER is depicted here as representative of ER and SR), which can be reduced by the chemical chaperone 4-phenyl butyrate (4-PBA). Prolonged ER/SR stress results in the activation of the Ca^2+^/calmodulin-dependent kinase II (CaMKII). CaMKII induces the protease calpain, resulting in protein degradation as well as cytoskeletal protein degradation (myolysis). The CaMKII-inhibitor hesperadin may ameliorate and reverse myolysis, e.g. calpain was found to degrade the microtubule network as well as contractile proteins. (3) Histone deacetylase 6 (HDAC6) is activated in AF resulting in deacetylation, and consequently the depolymerization of α-tubulin and loss of the microtubule network of the cardiomyocyte. HDAC inhibitors tubastatin-A, CKD-506 and ACY-1215 prevent excessive microtubule disruption and may also aid in recovery from AF. (4) AF causes DNA damage resulting in DNA repair by recruiting PARP1. Excessive PARP1 activation causes consumption of nicotinamide adenine dinucleotide (NAD^+^) to such an extent that NAD^+^ levels become depleted. NAD^+^ depletion leads to mitochondrial energy loss and an increase in reactive oxygen species (ROS) production and thus causes further DNA damage. The PARP inhibitors ABT-888, olaparib and nicotinamide riboside (NR) prevent excessive PARP1 activation and thereby attenuate the vicious cycle. ABT-888 and NR also accelerate cardiomyocyte recovery from AF. (5) Mitofusin 2 (MFN2) is a tether protein that connects the microtubule to the ER/SR and mitochondria. By pharmacological administration of tubulin stabilizers such as tubacin, taxol, or the ketone body β-hydroxybutyrate (βOHB), loss of contact can be prevented, resulting in the attenuation of AF promotion. Furthermore, the inhibitor of the mitochondrial calcium uniporter (MCU) Ru265 may positively influence mitochondrial function.

Proteins are complex macromolecules that are involved in the absolute functioning of cells such as atrial cardiomyocytes. Almost 14,000 different proteins on ribosomes are expressed in an average human cardiomyocyte, representing 73% of the total human proteins [10]. Protein quality control (PQC) systems monitor protein synthesis, maturation, function transport and breakdown (i.e., proteostasis) to ensure proteome integrity [11,12]. Chaperones play a crucial role within the PQC, especially the stress-related heat shock proteins (HSPs), as they are involved in the folding of proteins [13,14,15], and the protein degradation pathways, including the ubiquitin–proteasome system and autophagic protein degradation system [16,17]. Damaged, irreversibly misfolded and expired proteins are removed via protein degradation pathways with the ultimate goal of preventing toxic protein aggregation that may lead to dysfunction of the cardiomyocytes. During an AF episode, rapid fibrillation in atria induces cytoskeletal protein damage and consequently PQC system failure. PQC insufficiency then leads to the activation of the stress response, increased reactive oxygen species (ROS) as well as oxidative proteins and DNA damage [9]. Under normal physiological conditions, specific small HSPs, including highly expressed HSPB1, localize on the microtubule network and contractile proteins in cardiomyocytes, which thereby conserve the cardiomyocyte structure and electrophysiological and contractile functions [6,18,19]. During persistant AF, human HSPB1 expression levels reache their maximum in atrial tissue samples (Figure 1) [19]. In line with this, genetic and pharmacological boosting of HSP expression, with geranylgeranylacetone (GGA) or BGP-15, attenuated electropathology and AF promotion in various experimental AF model systems (Table 1) [6,20,21]. These findings indicate the exhaustion of HSP levels as a notable contributor to electropathology and a driver of AF.

### 2.2. Endoplasmic Reticulum Stress and Excessive Autophagic Protein Degradation

The rapid activation rate during AF causes a great burden to the cytoskeletal structure, leading to the degradation of sarcomeric and cytoskeletal proteins. Emerging evidence indicates that the excessive activation of autophagic protein degradation is an important contributor to cytoskeletal degradation [22,23]. Autophagy, an evolutionarily conserved protein degradation pathway, engulfs damaged and misfolded proteins into autophagosomes, followed by their lysosomal degradation. Autophagy enables elementary units such as amino acids and fatty acids to be recycled into ATP [24]. Although autophagy underlies vital cellular processes, excessive activation of the autophagic protein degradation pathway, as observed in AF, may trigger the degradation of cytoskeletal protein networks and impair calcium handling, in addition to electrical and contractile activities (Figure 1) [22,25]. Interestingly, the endoplasmic/sarcoplasmic reticulum (ER/SR) stress response acts as an initial trigger for autophagic protein degradation through the so-called unfolded protein response (UPR). The UPR further triggers the phosphorylation of the ER/SR stress sensor eIF2α at position S31, leading to the inhibition of general protein translation and at the same time initiates the selective expression of stress-responsive transcripts, including activating transcription factor 4 and 6 (ATF4 and ATF6, respectively) [26]. Furthermore, ATF4 and ATF6 signaling increases the C/EBP homologous Protein CHOP and autophagy protein expression and activation. Autophagic proteins include autophagy gene 12 (ATG12), microtubule-associated protein 1A/B light chain 3B and HSPA5, which together fuel the elongation of autophagosomes and activation of lysosomal activity [27].

One of the proteins involved in ER/SR proteostasis, as well as AF promotion, is Ca^2+^/calmodulin-dependent kinase II (CaMKII) (Figure 1) [28,29,30,31,32,33,34]. ER/SR stress results in increased intracellular Ca^2+^ and may thus promote Ca^2+^ overload, and consequently CaMKII activation [35,36]. Inhibition of CaMKII has shown to reduce the activity of the protease calpain [7,18,37] and may thus attenuate cytoskeletal protein degradation in AF [37,38,39]. In a mouse model, the CaMKII-δ inhibitor hesperadin alleviated the effects of cardiac ischemia, which can cause AF, and consequently presents an interesting target for AF treatment [32]. In AF, the ER stress-induced activation of autophagy constitutes an important mechanism. Blocking ER stress by the chemical chaperone 4-phenyl butyrate (4-PBA), attenuation of the overexpression of the ER chaperone HSPA5 or the mutated ER stress sensor eIF2α, inhibit the activation of autophagy, and thereby prevent electrical and contractile dysfunction (Table 1) [22].

These findings suggest a role for ER/SR stress-induced excessive autophagic protein degradation as a prominent modulator in the electropathology of AF.

### 2.3. Histone Deacetylase 6-Induced Microtubule Disruption

An intact and functional sarcomeric cytoskeleton network is of pivotal importance for the maintenance of balanced communication between different components of the proteostasis network and proper cardiomyocyte function [40,41]. The cytoskeleton network provides communication between various organelles and contractile proteins. It consists of actin filaments, intermediate filament proteins and microtubules. The cytoskeleton network interacts with membrane-associated proteins such as desmosomes and connexins, sarcomeric proteins, the nuclear envelope and various other organelles [40]. The sarcoplasmic cytoskeletal network ensures mechanical contractions and signal transductions between organelles, such as mitochondria, the nucleus, ER and SR. In addition, the cytoskeletal network assists in the transport of ubiquitinated proteins inside the proteostasis network and secures the architecture and shape of the cardiomyocyte. The cytoskeleton network depicts the backbone of the cardiomyocyte structure and function. During AF, loss of this cytoskeletal network was precipitated by the activation of histone deacetylase 6 (HDAC6) (Figure 1). HDAC6 deacetylates and depolymerizes α-tubulin, and in this capacity, the microtubule network becomes susceptible to degradation by the protease calpain, which becomes activated when the intracellular Ca^2+^ level is overloaded [7]. Interestingly, a dominant negative mutation in the α-tubulin catalytic domain of HDAC6 conserved the electrical and contractile function of the cardiomyocyte and the microtubule network [7]. Therefore, inhibition of HDAC6 might represent a valuable target for AF treatment. These selective HDAC6 inhibitors exemplarily include tubastatin-A, CKD-506 and ACY-1215 [7,42,43]. While CKD-506 and ACY-1215 have been investigated in the context of immunology and oncology, tubastatin-A protects against tachypacing-induced loss of the microtubule network, electrical abnormalities and AF vulnerability in various experimental model systems [7]. Furthermore, AF-induced loss in contacts between the microtubule network with the SR, ER and mitochondria via the tether protein mitofusin 2 (MFN2) results in Ca^2+^ loss in organelles, UPR in the ER and mitochondrial dysfunction, and therefore drives AF [44]. Additionally, the loss of contact between these organelles may potentially be initiated by spontaneous SR Ca^2+^ releases. The Ca^2+^-induced ectopic activity may then trigger AF [45,46]. Interestingly, Ca^2+^ overload in the cytosol of atrial cardiomyocytes activates calpain, which then leads to sarcomeric, ionchannel and cytoskeletal protein cleavage, and consequently drives AF promotion [37,47].

Collectively, the results emphasize a key role for HDAC6 activation in AF-related structural damage and electropathology, providing suggestions for a novel therapeutic target in AF.

### 2.4. NAD^+^ Depletion Due to DNA Damage-Induced PARP1 Activation

Conservation of the integrity of the proteostasis network is a continuous, energy-intensive process in cardiomyocytes [15]. Moreover, the rapid activation rate during AF will additionally challenge the capacity of energy production in mitochondria. Suboptimal mitochondrial energy production features an increase in ROS production as well as oxidative protein and DNA damage [48,49,50]. Indeed, in AF, this sequela is supported by emerging evidence (Figure 1) [44,51]. Recent findings revealed that the electropathology in AF is precipitated by excessive activation of the DNA repair machinery. Oxidative DNA damage induces poly(ADP)-ribose polymerase 1 (PARP1) activation [9]. Due to the synthesis of PARP1 ADP ribose chains, the cellular levels of mitochondrial nicotinamide adenine dinucleotide (NAD^+^) are depleted. Thereby, energy loss is augmented and ROS formation further triggers oxidative DNA damage and electropathology. Interestingly, NAD^+^ supplementation or depletion of PARP1 by pharmacological or genetic means precludes electrical and contractile loss in function. In experimental model systems for AF, the inhibition of PARP1 with ABT-888, olaparib or nicotinamide riboside protected against contractile dysfunction by preventing NAD^+^ depletion and oxidative stress and DNA damage as a result (Table 1). Consistent with these findings, the cardiomyocytes of patients with persistent AF also show significant DNA damage, which correlates with PARP1 activity. In addition, in heart tissue, DNA damage was associated with electrophysiological deterioration, a reduction in excitability on the cardiomyocyte level and increased action potential dispersion. These molecular and structural alterations function as a substrate for further electropathology and AF promotion [9,52]. These findings not only indicate a novel mechanism by which AF hampers cardiomyocyte function, but also propose PARP1-inhibition and/or NAD^+^ supplementation as a possible therapeutic target that may preserve the proteostasis network and cardiomyocyte function in clinical AF.

### 2.5. Mitochondrial Dysfunction

Mitochondria represent almost one third of the myocardial volume and are defined as the powerhouses of the cell, as they play a pivotal role in maintaining the energetic and metabolic homeostasis in cardiomyocytes [53]. Via oxidative phosphorylation, mitochondria synthesize the high-energy molecule adenosine triphosphate (ATP), which is essential for mechanical and electrical activities of the heart. Therefore, myocardial contraction is tightly coupled to ATP synthesis. For ATP generation, various molecules present suitable energy substrates in the heart, including fatty acids, glucose, amino acids, and ketone bodies [54]. Under normal physiological conditions, cardiomyocytes mainly utilize fatty acids (60–80%) and carbohydrates (20–40%) as their predominant substrates for oxidative phosphorylation [55]. The by-product of ATP production is the formation of oxygen superoxides or ROS [56]. The formed ROS is neutralized by an endogenous scavenging system. However, excessive ROS production and an impaired scavenging system can cause oxidative stress in cardiomyocytes and promote dysfunction, including AF [57,58].

A recently published study uncovered the role of mitochondrial dysfunction as a driver of AF [44]. In an experimental model system, HL-1 atrial cardiomyocytes progressively showed upregulated mitochondrial HSPs and impairment of mitochondrial Ca^2+^ handling when tachypaced. Moreover, the measured decrease in the mitochondrial membrane potential, respiration and ATP production indicate increased mitochondrial dysfunction. Comparable features of mitochondrial dysfunction could also be observed in atrial biopsies from AF patients, namely aberrant ATP levels, induced mitochondrial HSP expression and a fragmented mitochondrial network [44]. Interestingly, AF promotion could be attenuated by targeting the mitochondrial calcium uniporter (MCU) through partial blocking or downregulation. While Ru265 was shown to be a potent MCU inhibitor and inhibitor of hypoxia-induced mitochondrial dysfunction [59], effectiveness in an AF model needs to be elucidated. SS31, a compound that improves bioenergetics in mitochondria, has revealed to be protective against mitochondrial stress and mitochondrial and contractile dysfunction upon tachypacing in experimental AF model systems (Figure 1) [44].

As is mentioned above, emerging evidence indicates a role for the loss of contact between the ER/SR and mitochondria to drive experimental and clinical AF [60]. The stability of ER/SR and mitochondrial contact is regulated by microtubules and the ER/SR-mitochondrial tether protein MFN2. Conservation of the ER/SR-mitochondrial contacts with the microtubule stabilizers tubacin, taxol, or the ketone body β-hydroxybutyrate (βOHB) attenuates the loss of contact and improves mitochondrial function (Table 1) [60]. These findings substantiate the importance of the microtubule, ER/SR and mitochondrial network; loss of contact in this network results in myocardial oxidative stress [58], unfolded protein responses in the ER and mitochondrial dysfunction [40,50,51,52,53]. Conservation of this network is of utmost importance for balanced intracellular communication in the proteostasis network in order to ensure proper contractile function of the cardiomyocyte.

Together, these results show that conservation of the mitochondrial function protects against electropathology and AF promotion and identifies this organelle as a potential novel therapeutic target.

**Table 1:** Overview of the current drug treatments and prospective drugs in AF treatment.

Key Modulators	Action	Drug	Clinical Phase	Indication	Reference
HSP	HSP induction	GGA (teprenone)	IV	Gastric ulcers	[61]
				Gastric lesion	[62]
			Preclinical	AF	[6]
			II	Heart failure with preserved ejection fraction	[63]
			II	Cardiac bypass surgery	[64]
	HSP induction	BGP-15	Preclinical	Duchenne muscular dystrophy	[65]
			Preclinical	AF	[21]
	Induction of IGF1R phosphorylation		Preclinical	AF and heart failure	[66]
	JNK inhibitor		II	Insuline resistance	[67]
	PARP inhibitor		Preclinical	Cardioprotective	[68]
	HSP induction	GGA derivatives	Preclinical	AF	[18,69]
ER stess	Chemical chaperone	4-PBA	II	Amyotrophic lateral sclerosis	[70]
		(Buphenyl^®^ and Ammonaps^®^)	II	Huntington’s disease	[71]
			II	Pulmonary tuberculosis	[72]
			III	Maple syrup urine disease	[73]
			IV	Diabetes	[74]
			II	Urea cycle disorder	[75]
			Preclinical	AF	[22]
	CaMKII-δ inhibitor	Hesperadin	Preclinical	Cardiac ischemia	[32]
HDAC6	HDAC6 inhibitor	Tubastatin A	Preclinical	AF	[7]
		CKD-506	Preclinical	Multiple sclerosis rheumatoid arthritis	[76,77]
		ACY-1215 (ricolinostat)	II	Lymphoma	[78]
DNA damage	PARP inhibitor	ABT888	II	Metastatic breast cancer	[79]
			II	Hepatocellular carcinoma	[80]
			I	Adult solid neoplasm	[81]
			II	Ovarian cancer	[82]
			II	Colorectal cancer	[83]
			Preclinical	AF	[9]
		Olaparib	Preclinical	Ovarian cancer with BRCA 1/2 mutation	[84]
Mitochondrial dysfunction	PARP inhibitor	Nicotinamide (niagen^®^)	III	Lung carcinoma	[85]
	Sirt inhibitor		III	Chronic kidney disease	[86]
	NAD-precursor		II	Neurodegenerative disease	[87]
			II	Alzheimer’s disease	[88]
			Preclinical	AF	[7]
	Cardiolipin binder	SS31 (MTP-131, elamipretide)	II	Mitochondrial myopathy	[89]
			II	Chronic heart failure	[90]
			II	Age-related macular degeneration	[91]
			II	Reperfusion injury STEMI	[92]
			III	Barth syndrome	[93]
			Preclinical	Friedreich ataxia	[94]
			Preclinical	AF	[44]
	MCU inhibitor	Ru265	Preclinical	Hypoxia-induced mitochondrial dysfunction	[59]

AF—atrial fibrillation; BRCA—BReast Cancer; GGA—geranylgeranylacetone; HDAC6—histone deacetylase 6; HSP—heat shock protein; MCU—mitochondrial calcium uniporter; NAD—nicotinamide adenine dinucleotide; PARP—poly(ADP)-ribose polymerase 1; 4-PBA—4-phenyl butyrate.

## 3. Recovery from Proteostasis Derailment

Unfortunately, the current AF therapy fails in preventing AF progression [3,4,95]. Therefore, treatments directed at recovery from electropathology are clinically highly relevant. In order to address AF recovery, a deeper understanding of the molecular mechanisms driving the recovery from electropathology is required. Thus novel druggable targets that may accelerate this process and ultimately enhance AF free episodes in clinical AF shall be identified.

### 3.1. Recovery from Electropathology in Animal Models for AF: A Slow Process

During AF, deterioration of the structural and electrical integrity has been observed in experimental and clinical AF [96]. Several research groups studied recovery from AF-induced structural damage in large animal models. Ausma et al. used goats as an animal model to observe the reversion of AF-induced structural damage over a recovery period of several months [97]. Mahajan, Rajiv et al. investigated the relation between spontaneous AF and structural damage in the context of obesity and weight-loss in a sheep model [98]. Furthermore, Everett et al. utilized a chronic AF dog model with moderate mitral valve regurgitation, followed by pacing in the sinus rhythm and recovery from structural damage [99] (Table 2).

In all animal models, AF induced structural and electrical changes. During the recovery period in normal sinus rhythm, prior identified electrical changes were reversed, but increased AF inducibility persisted in all experimental model systems [97,98,99]. This indicates that other molecular mechanisms underlie AF susceptibility. Of note, the majority of proteins expressed in atrial cardiomyocytes are cytoskeletal proteins [100]. As cytoskeletal proteins play a key role in contractility, their loss of function and degradation will lead to prolonged structural damage [97] and may underlie AF vulnerability. Upon AF induction, changes in the expressions of the cytoskeletal proteins including cardiotin, titin, desmin and α smooth muscle actin (αSMA) were observed [97]. After a recovery period of several weeks to months, the expression levels only partly recovered [97]. Reversible changes in the expression of gap-junctional connexins (Cx) were observed [97,98]. AF induction also resulted in increased αSMA-positive cells, which did not revert back to normal but slightly decreased with recovery [97]. αSMA is a marker for transdifferentiated myofibroblasts and is consequently connected to fibrosis [101]. In AF dog models, as well as in obese sheep, fibrosis was observed, regressing over a sufficient recovery period [98,99]. Mitochondria size, shape and number were found to be altered with the loss of cristae definition [97,99] and cardiomyocyte shape as well as diameter differed from the controls [97,99]. The cardiomyocyte diameter depends on the extent of AF stress-induced cytoskeletal disruption (so-called myolysis) [19,102], adeterioration steadily progressing over AF induction [103]. After a recovery period, the degree of myolysis was partly normalized and cytoskeletal disruption remained [97,99]. As myolysis is characterized by sarcoplasmic reticulum and contractile protein loss, the contractile force was diminished and cardiomyocytes were enlarged [102].

### 3.2. Proteoceuticals to Accelerate Recovery: HSP-Inducing Compounds

As HSP exhaustion is a key driver of AF promotion, the normalization of HSP levels may aid in the recovery from AF. Of the HSP-inducing compounds available, geranylgeranylacetone (GGA) and GGA-derivatives have been tested for their potential to reverse AF-induced structural damage in HL-1 atrial cardiomyocytes [18,69]. In this AF-recovery model, HL-1 cardiomyocytes were tachypaced for 10 h, which resulted in a significant and continuous loss in Ca^2+^ transients.This observation coincided with microtubule disruption and inhibition of gene transcription and translation of contractile proteins. Post-treatment with GGA and GGA-59 significantly enhanced HSP expression, restored the microtubule network, enhanced gene expression and resulted in the normalization of Ca^2+^ transients [18], indicating that HSP-inducing compounds may accelerate recovery from electropathology in experimental and potentially also clinical AF (Figure 1).

In addition to GGA, the HSP-inducing compound BGP-15 was found to regenerate contractile function in skeletal muscle [104]. A favorable effect on cardiomyocytes can be suggested. Further studies are warranted to investigate the BGP-15-induced recovery effects from AF.

### 3.3. NAD^+^ Supplementation as a Proteoceutical to Enhance Cardiomyocyte Recovery

Another druggable target to enhance recovery from electropathology and AF may be the conservation of NAD^+^ levels. NAD^+^ is a coenzyme that is essential for numerous cellular processes, such as ATP generation, ROS detoxification and NAD^+^ as an energy substrate for PARPs to create PAR chains [58,105]. Pharmacological inhibition of PARP1 activity with ABT888 has been shown to be cardioprotective in AF patients [9]. Interestingly, post-treatment of tachypaced HL-1 atrial cardiomyocytes with ABT888 attenuated PARP1 activity, resulting in an increase in mitochondrial NAD^+^ levels, attenuation of ROS production and consequently normalization of Ca^2+^ transients and cardiomyocyte function. Thus, PAPR1 inhibition by ABT-888 conserves NAD^+^ levels and may result in accelerated recovery of atrial cardiomyocyte function after AF (Figure 1) [9].

Recently, a prospective study, i.e., the HF-AF ENERGY trial, was published [106]. In this trial, patients with AF and heart failure with reduced ejection fractions were treated with the precursor of NAD^+^, nicotinamide riboside (NR). Following a 4-month period of observation, 4 months of intervention with NR was performed. The primary outcome was a reduction in the AF burden. As a secondary outcome, the study determined the recovery of mitochondrial function and energy metabolism.

Studying the effects of proteoceuticals in clinical AF may enhance our insights into effective AF recovery compounds. Therefore, further studies addressing AF recovery are essential.

### 3.4. Additional Proteoceutical Intervention Approaches for AF Recovery

Another potential target for recovery from AF is ER/SR proteostasis. Pre- as well as post-treatment with the CaMKII-δ inhibitor hesperadin alleviated hypoxic consequences in ischemia/reperfusion (I/R) injury [31,32]. Hesperadin, and thus CaMKII-δ inhibition, could be an interesting target for the reversion of hypoxia-induced AF, despite their off-target potential.

Histone deacetylase 6 derailment promotes AF induction by microtubule destabilization, and thus contractile dysfunction [7,107]. In a Drosophila model, Alzheimer’s phenotypical tau-induced microtubule defects were recovered by ACY-1215 and tubastatin A mediated HDAC6 inhibition. Interestingly, these compounds also attenuated the already formed defects, including microtubule network disruption [43]. Recovery in the context of cardiomyocytes and AF should thus be elucidated (Table 3).

## 4. Conclusions

AF is the most common form of tachyarrhythmia and a leading cause of stroke and heart failure. Unfortunately, current treatments are not directed at the underlying molecular mechanisms that drive electrical conduction abnormalities and AF. The degree of this so-called ‘electropathology’ corresponds to the response to anti-AF treatment. Therefore, unravelling the molecular mechanisms underlying electropathology may lead to the development of directed therapies for the prevention of AF onset and recurrence. The derailment of proteostasis has been identified as a key molecular mechanism for electropathology. Importantly, several proteoceuticals have been found to attenuate AF, and some have been tested so far for their potency to accelerate recovery from electropathology. As acceleration from electropathology is clinically highly relevant, future research that investigates this concept in greater detail in order to prevent AF recurrence and consequently ameliorate this aspect of AF management is required.

## Figures and Tables

**Table 2:** Observations of electropathology during recovery from AF in three different large animal models. The direction of the arrows mark whether observations were up or downregulated. The equals symbol significates no detected changes. Italics highlight alterations that could still be observed post recovery period.

Context [Ref.]	Induction of AF	Model (Number ^a^)	Tim Course ^c^	Electrical Change Observations/*Present Post Recovery*	Structural Changes Observations/*Present Post Recovery*				
					Myocytes	Structural Proteins	Sarcomeres	Mitochondria	Fibrosis
Remodeling in AF [97]	Implanted pacemaker	Goat (18)	4 months AF + 4 months recovery	AFCL length ↓ *AF inducibility* ↑	*Myolysis* Diameter ↑ Size↑	Cx-40 ↓ Cx-43 = Cardiotin ↓ *Titin* ↓ *Desmin* ↓	*Partially destroyed*	*Elongated* *Size* ↓	-
Obesity [98]	Spontaneous	Sheep (30)	36 weeks weight induction + 32 weeks weight reduction ^b^	AERP duration ↓ *AF inducibility* ↑ Velocity ↓ Heterogeneity ↑	-	Cx-43↓	-	-	Fibrosis ↑
Remodeling in AF [99]	Implanted pacemaker and moderate mitral valve regurgitation	Dog (8)	8 weeks AF + 2 weeks recovery	AERP duration ↓ AFCL length ↓ *AF Inducibility* ↑	*Myolysis* *Diameter* ↓	-	*Partially destroyed*	*Number* ↑ *Size* ↑	TGFβ ↑ Fibrosis ↑

^a^—without controls; ^b^—weight following weight reduction was higher than control; ^c^—maximal endpoints; AERP—atrial effective refractory period; AF—Atrial Fibrillation; AFCL—atrial fibrillation cycle length.

**Table 3:** Drugs with potential revertive effects on AF.

Key Modulators	Mode of Action	Drug	Observation	Context of Observation	Model	Reference
HSP	HSP Induction	BGP-15	BGP-15 accelerated recovery of muscle function after muscle injury	Ischemia reperfusion injury of soleus muscle	Mice	[104]
		GGA*59	Tachypaced cells post-treated with GGA*59 widely restored calcium transient loss	AF	HL1 cardiomyocytes	[69]
DNA damage	PARP inhibitor	ABT-888	PARP1 inhibition accelerates post-TP recovery	AF	HL-1 cardiomyocytes	[9]
		Nicotinamide riboside	Prospective study	Effect of nicotinamide riboside on the AF burden of patients with HF (pre- and post-treatment)	Humans	[106]
ER stress	CaMKII-δ inhibitor	Hesperadin	CaMKII-δ inhibitor post-treatment alleviates ischemic consequences and protects functional integrity	Cardiac ischemia, reperfusion injury	Mice	[32]
HDAC6	HDAC6 inhibitor	ACY-1215 tubastatin-A	HDAC6 inhibitors ameliorated formed tau-induced microtubule defects	Alzheimer’s disease	*Drosophila*	[43]

AF—atrial fibrillation; GGA* 59—geranylgeranylacetone derivative 59; ER—endoplasmic reticulum; HDAC6—histone deacetylase 6; HL-1—cardiac muscle cell line; HSP—heat shock protein; PARP—poly(ADP)-ribose polymerase 1.
